# RASopathies and cardiac manifestations

**DOI:** 10.3389/fcvm.2023.1176828

**Published:** 2023-07-17

**Authors:** Nazia Hilal, Zi Chen, Ming Hui Chen, Sangita Choudhury

**Affiliations:** ^1^ Division of Genetics and Genomics, Department of Pediatrics, Boston Children’s Hospital, Boston, MA, United States; ^2^ Harvard Medical School, Boston, MA, United States; ^3^ Broad Institute of Harvard and MIT, Cambridge, MA, United States; ^4^ Department of Surgery, Brigham, and Women’s Hospital, Boston, MA, United States; ^5^ Department of Cardiology, Boston Children’s Hospital, Boston, MA, United States

**Keywords:** rasopathy, cardiac abnormalities, congenital heart disease, hypertrophic cardiomyopathy, ras/MAPK

## Abstract

As binary switches, RAS proteins switch to an ON/OFF state during signaling and are on a leash under normal conditions. However, in RAS-related diseases such as cancer and RASopathies, mutations in the genes that regulate RAS signaling or the RAS itself permanently activate the RAS protein. The structural basis of this switch is well understood; however, the exact mechanisms by which RAS proteins are regulated are less clear. RAS/MAPK syndromes are multisystem developmental disorders caused by germline mutations in genes associated with the RAS/mitogen-activated protein kinase pathway, impacting 1 in 1,000–2,500 children. These include a variety of disorders such as Noonan syndrome (NS) and NS-related disorders (NSRD), such as cardio facio cutaneous (CFC) syndrome, Costello syndrome (CS), and NS with multiple lentigines (NSML, also known as LEOPARD syndrome). A frequent manifestation of cardiomyopathy (CM) and hypertrophic cardiomyopathy associated with RASopathies suggest that RASopathies could be a potential causative factor for CM. However, the current supporting evidence is sporadic and unclear. RASopathy-patients also display a broad spectrum of congenital heart disease (CHD). More than 15 genes encode components of the RAS/MAPK signaling pathway that are essential for the cell cycle and play regulatory roles in proliferation, differentiation, growth, and metabolism. These genes are linked to the molecular genetic pathogenesis of these syndromes. However, genetic heterogeneity for a given syndrome on the one hand and alleles for multiple syndromes on the other make classification difficult in diagnosing RAS/MAPK-related diseases. Although there is some genetic homogeneity in most RASopathies, several RASopathies are allelic diseases. This allelism points to the role of critical signaling nodes and sheds light on the overlap between these related syndromes. Even though considerable progress has been made in understanding the pathophysiology of RASopathy with the identification of causal mutations and the functional analysis of their pathophysiological consequences, there are still unidentified causal genes for many patients diagnosed with RASopathies.

## Introduction

**Figure 1:**
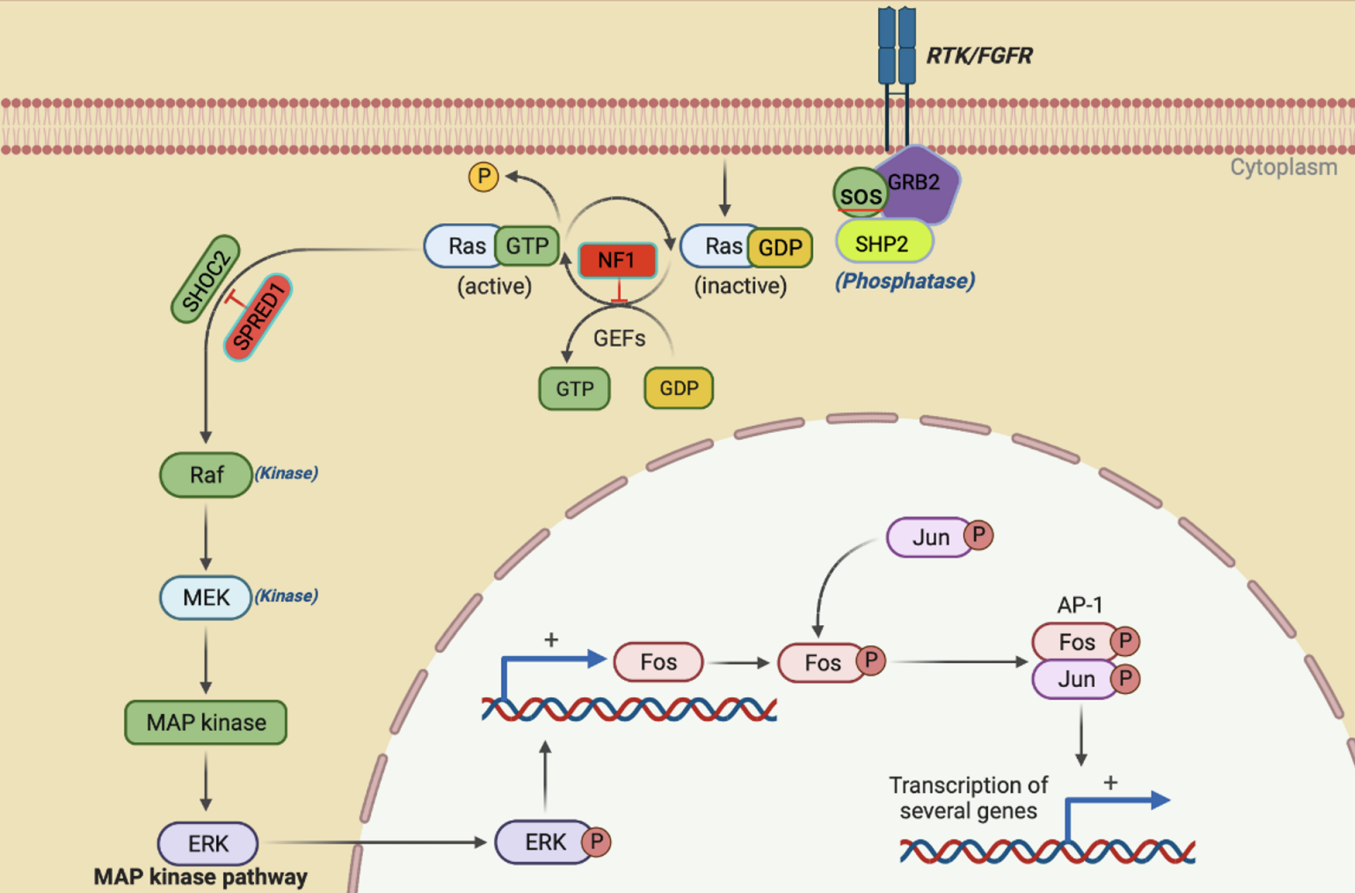
The activation of RAS/ERK occurs when cell survival signals bind to receptor tyrosine kinases (RTK). Once the RTK intracellular domain is phosphorylated following this binding, it triggers a sequence of events that ultimately results in the activation of RAS. NF1 and SPRED 1 act as negative effectors of the pathway. The activation of RAS recruits and activates RAF, which is the first MAPK in this pathway. Then, RAF phosphorylates and activates MEK1/2, which finally activates ERK1/2 through dual phosphorylation on tyrosine and threonine. ERK1/2 then goes on to activate various substrates downstream like FOS and JUN that ultimately leads to transcriptional activation of genes involved in cell proliferation and survival.

Rat Sarcoma Virus, a highly conserved protein, belongs to a class of proteins called small GTPase. The three most widely studied RAS genes in humans are HRAS, KRAS, and NRAS, named after the Harvey Rat sarcoma virus, Kirsten Rat sarcoma virus, and NRAS, for its initial identification in neuroblastoma cells. Since the identification of the RAS protein in 1982, extensive studies have been conducted to identify the RAS-associated pathway and its involvement in human disease. RASopathies refer to multisystem disorders caused by gene mutations that belong to the RAS/MAPK (Mitogen-activated protein kinase) signaling pathway. RAS can be either “switched on” or activated by incoming signals through growth factors binding to receptor tyrosine kinases (RTKs), G-protein-coupled receptors, cytokine receptors, and extracellular matrix receptors, or activated by mutations in RAS genes, which can lead to the production of permanently activated RAS proteins and can cause unintended and overactive signaling inside the cell, even in the absence of incoming signals. The mitogen-activated protein kinase (MAPK) pathway is one of RAS's critical downstream signaling cascades. Activated RAS leads to the phosphorylation of Raf, leading to the activation of the MAPK kinases MEK1 and/or MEK2; these, in turn, phosphorylate and activate ERK1 and/or ERK2. ERK1 and ERK2 are the ultimate effectors which exert their function on many downstream molecules in the cytoplasm and nucleus (Figure 1). RASopathy disorders include wide range of disorders such as neurofibromatosis type 1, Noonan syndrome, Noonan syndrome with multiple lentigines, Costello syndrome, cardio-facio-cutaneous syndrome, and Legius syndrome (1, 2), exhibiting multi-organ dysfunction, including craniofacial dysmorphology, cardiac malformation, cutaneous, musculoskeletal, and ocular abnormalities, neurocognitive impairment, hypotonia and increased cancer risk (1–3). In Table 1, we summarize the critical and cardiac-specific features as well as all other RASopathy-associated malformations. This review will discuss only the cardiac manifestation in RASopathies associated with Noonan syndrome and Neurofibromatosis type 1 (NF1). RASopathy-related heart defects include congenital heart disease (CHD), hypertrophic cardiomyopathy (HCM) as well as dilated cardiomyopathy (DCM).

**Table 1:** Different features of RASopathies, including main features, cardiac-specific features, and critical cardiac features.

RASopathy	Main Feature		Cardiac specific Feature		Critical cardiac feature
Noonan syndrome	Short stature, webbed neck, low-set ears, widely spaced eyes, hypertelorism, pectus excavatum/carinatum, cryptorchidism, bleeding diathesis, lymphatic dysplasia		Pulmonary stenosis (PS), hypertrophic cardiomyopathy (HCM)		Atrial septal defect (ASD), ventricular septal defect (VSD), coarctation of the aorta (CoA), critical aortic stenosis (AS)
** **	Gene	Mutation Type	Mutation Location	Function	Cardiac Implications
Noonan syndrome	PTPN11	Missense, splice site	Exon 3, 7, 8, 12, 13	Encodes for SHP-2 protein which regulates RAS signaling pathway	Pulmonic stenosis (PS), hypertrophic cardiomyopathy (HCM), arrhythmias
	SOS1	Missense, frameshift, splice site, large deletions	Exon 2, 3, 5, 12, 13, 16, 17, 18, 19, 20, 21	Encodes for SOS1 protein which activates RAS signaling pathway	Pulmonic stenosis (PS), hypertrophic cardiomyopathy (HCM), cardiomyopathy, septal defects
	RAF1	Missense	Exon 7, 12, 14	Encodes for RAF1 protein which activates the MEK/ERK signaling pathway	Hypertrophic cardiomyopathy (HCM), pulmonary valve stenosis (PVS), cardiomyopathy
	KRAS	Missense	Exon 2	Encodes for KRAS protein which regulates cell division and differentiation	Hypertrophic cardiomyopathy (HCM)
RASopathy	Main Feature		Cardiac specific Feature		Critical cardiac feature
Cardio-facio-cutaneous syndrome	Short stature, sparse hair, curly hair, prominent forehead, hypertelorism, downward slanting palpebral fissures, ptosis, hearing loss, intellectual disability		Hypertrophic cardiomyopathy (HCM), pulmonary valve stenosis (PVS)		Atrial septal defect (ASD), ventricular septal defect (VSD), critical pulmonary valve stenosis (c-PVS), severe mitral valve disease (MVD)
** **	Gene	Mutation Type	Mutation Location	Function	Cardiac Implications
Cardio-facio-cutaneous syndrome	BRAF	Missense, frameshift, splice site, large deletions	Exon 5, 8, 11, 15	Encodes for BRAF protein which activates the MEK/ERK signaling pathway	Pulmonic stenosis (PS), hypertrophic cardiomyopathy (HCM), arrhythmias, cardiomyopathy
RASopathy	Main Feature		Cardiac specific Feature		Critical cardiac feature
Costello syndrome	Short stature, coarse facies, curly hair, loose skin, hypertrophic cardiomyopathy, feeding difficulties, intellectual disability, neoplasia		Hypertrophic cardiomyopathy (HCM), pulmonic stenosis (PS)		Atrial septal defect (ASD), ventricular septal defect (VSD), critical pulmonary valve stenosis (c-PVS), severe mitral valve disease (MVD)
** **	Gene	Mutation Type	Mutation Location	Function	Cardiac Implications
Costello syndrome	HRAS	Missense	Exon 2, 3	Encodes for HRAS protein which regulates cell division and differentiation	Hypertrophic cardiomyopathy (HCM), tachycardia, arrhythmias
RASopathy	Main Feature		Cardiac specific Feature		Critical cardiac feature
Legius syndrome	Café-au-lait macules, lipomas, macrocephaly, learning disabilities, Noonan-like facies		None reported		None reported
** **	Gene	Mutation Type	Mutation Location	Function	Cardiac Implications
Legius syndrome	SPRED1	Missense, frameshift, splice site, large deletions	Exon 1, 2, 3, 5, 6, 7, 8, 9	Encodes for SPRED1 protein which acts as a negative regulator of RAS signaling pathway	Pulmonic stenosis (PS), hypertrophic cardiomyopathy (HCM), valvular heart disease (VHD)
RASopathy	Main Feature		Cardiac specific Feature		Critical cardiac feature
Neurofibromatosis Type 1	Café-au-lait macules, neurofibromas, Lisch nodules, scoliosis, optic gliomas, learning disabilities, skeletal abnormalities		Pulmonary stenosis (PS)		Atrial septal defect (ASD), ventricular septal defect (VSD), hypertrophic cardiomyopathy (HCM)
** **	Gene	Mutation Type	Mutation Location	Function	Cardiac Implications
Neurofibromatosis Type 1	NF1	Missense, nonsense, frameshift, splice site, large deletions	Most commonly 17q11.2	Encodes for neurofibromin protein which acts as a negative regulator of RAS signaling pathway	Hypertrophic cardiomyopathy (HCM), pulmonary stenosis (PS), congenital heart defects (CHD)

### Noonan syndrome and cardiac manifestation

Noonan syndrome (NS1, OMIM 163950), caused by mutation and activation of the genes involved in the RAS-MAPK pathway, including *PTPN11, SOS1, KRAS, NRAS, RAF1, BRAF, RIT1, and LZTR1*, is a common developmental disorder with an autosomal dominant inheritance. The incidence is 1:1,000–2,500 live births. Many patients with NS1 indicate cardiovascular abnormalities, most commonly in the form of congenital heart diseases, such as pulmonary valve stenosis, septal defects, left-sided lesions, and complex forms with multiple anomalies. The most common congenital heart disease (CHD) involves pulmonary valve stenosis in 50%–60% of patients, and a small portion (6%–10%) indicates an atrial septal defect. The other CHDs, such as ventricular septal defect, atrioventricular canal defect, and aortic coarctation, are observed less frequently in NS1 patients (4–7). The second most prevalent cardiovascular anomaly associated with NS1 is HCM, present in approximately 20% of patients. Although NS1 is clinically heterogeneous and can manifest at any age, 80% of NS-1 HCM diagnoses are made early in infancy, and compared to non-syndromic types of HCM, NS1-HCM patients have a greater degree of ventricular hypertrophy, a higher prevalence, and a more severe pattern of left ventricular outflow tract obstruction (LVOTO). A literature survey indicates that a patient's likelihood of NS1-HCM varies significantly according to the gene mutated in the RAS-MAPK pathway. A few studies suggest an association between DCM and NS1 to some extent, where histology/echocardiography showed hypertrophy of myocardial fibers with focal interstitial fibrosis with no evidence of myocardial disarray. The features were consistent with DCM (8–11).

Noonan syndrome with multiple lentigines (NSML), which is also known as LEOPARD syndrome, has the cardiac manifestation of pulmonary valve stenosis and hypertrophic cardiomyopathy along with brown spots on the skin called lentigines, caused by the mutation in one of four genes: BRAF, MAP2K1, PTPN11, and RAF1 (12–14).

### Genes involved in Noonan syndrome

**Figure 2:**
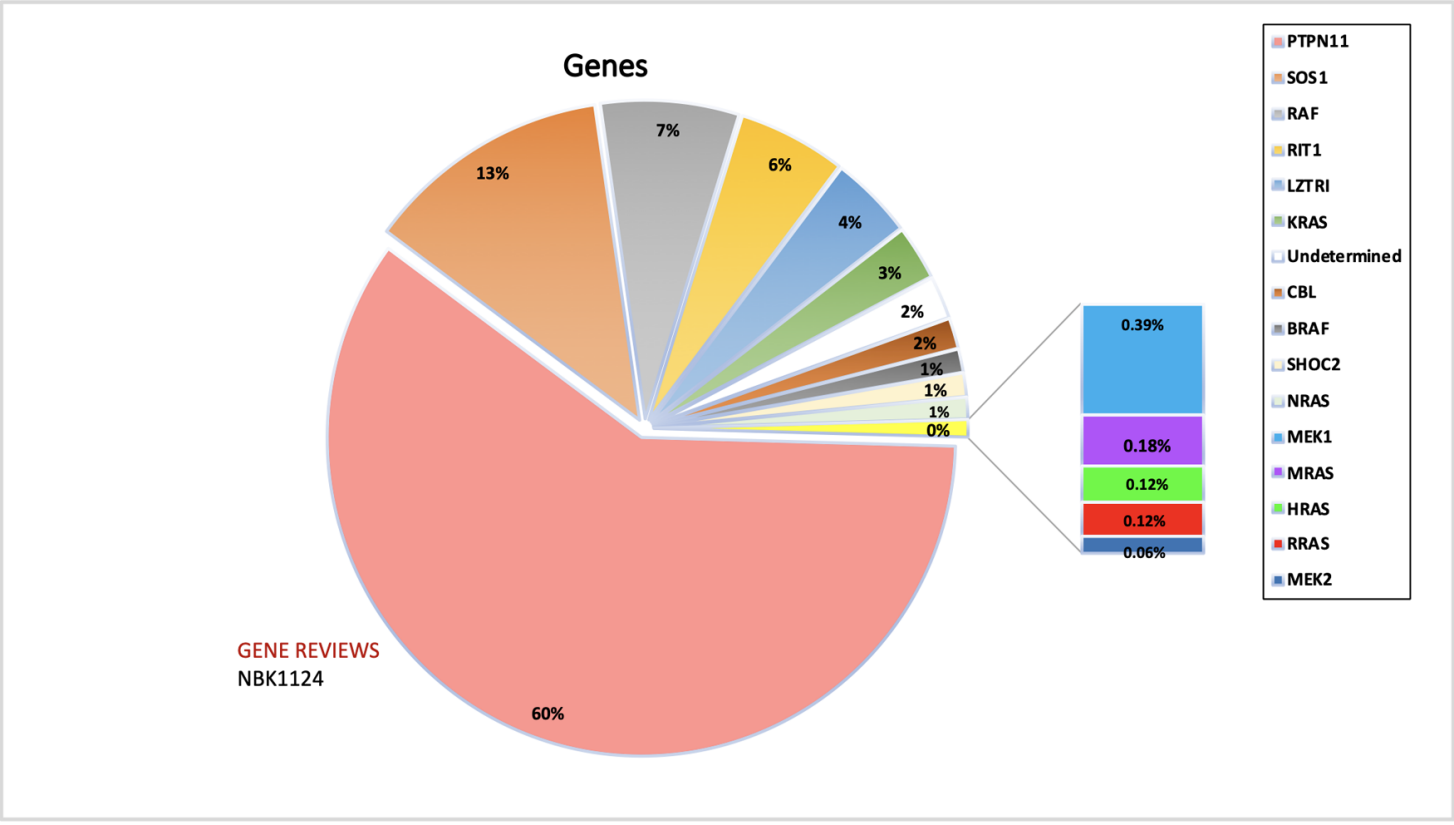
The figure represents the information of the genes implicated in NS and associated RASopathies. PTPN11, SOS1 and RAF alone makes up for more than 90% of the total pathogenic mutations. The data is obtained from the NSEuroNet database.

*PTPN11* was found to be the most studied gene in NS populations (29 studies vs. 16 studies or fewer for other genes). Possible reasons include that *PTPN11* was the first gene of the RAS/MAPK pathway to be implicated in NS in 2001, while *KRAS* was discovered only five years later, followed by SHP-2. Although *KRAS* was already involved in malignancy disorders through various somatic mutations, its interrelation with NS was found *via* germline mutations in 2006 (15). Subsequently, in 2007, *SOS1, RAF1*, and *MAP2K1* genes were found to be implicated in NS (16), after which *BRAF* (12, 16), *NRAS* (17), and *RIT1* (18) (RAS/MAPK kinase genes) were shown to be involved. Notably, the chronology of discovering the involved genes does not correlate with their frequency or the intensity of the phenotypic manifestations but is merely incidental. The most common gene implicated in the causation of NS is still *PTPN11* (60%), constituting 52.6% of all mutations detected in Noonan patients to date (Figure 2). The second most found mutated gene in NS is *SOS1*(16.4% of patients). Furthermore, *RIT1* and *RAF1* have been found to have a prevalence of 8%, making them the third most-involved genes. Therefore, mutations in *PTPN11, SOS1, RAF1, and RIT1* alone comprise 93% of the mutations causing NS. Hence, these genes are included in the first line of genetic screening in patients with the NS phenotype. Table 2 summarizes the genes and domains involved in RASopathies. *KRAS* (2.8%) and *NRAS* (0.8%) have the lowest incidence among all reported cases of Noonan syndrome caused by the RAS subfamily of genes involved in the RAS/MAPK pathway, in contrast with the *RIT1* gene in the same family. Similarly, *BRAF* constitutes 2.3% among RAF family members compared to the more prevalent *RAF1*. Table 1 emphasizes the genes involved in different RASopathies and their normal function, mutation type, mutation location, and cardiac implications.

**Table 2:** Common RASopathy-associated mutations.

Component	Gene	Mutation	Domain	Type
Phosphatases	*PPP1CB*	p.P49R	N-terminus	NS
	*PTPN11*	p.D61G/N/H	N-SH2	NS
		p.Y63C		
		p.Q79R/P		
		p.N308D/S/T	Phosphatase	
		p.Y279C/S		NS-ML
		p.T469M/P		
RAS isoforms	*HRAS*	p.G12S/A/C	RAS	CS
	*KRAS*	p.V14I		NS
		p.P34R/K		
		p.I36M		
		p.T58I		
		p.G60R/S		
		p.D153V		
	*NRAS*	p.G60E		
	*MRAS*	p.G23R/V		
	*RRAS*	p.G23dup		
	*RIT1*	p.A57G		
		p.F82l/I/V		
		p.M90I		
		G95A		
Kinases	*BRAF*	p.Q257R/K	CRD	CFC
		p.L597V	Kinase	
	*RAF1*	p.S257l/K/P	CR2	NS
	*MEK1*	p.Y130C/N/H	Kinase	CFC
	*MEK2*	p.F57C/I/V/l	N-terminus	
GEFs	*SOS1*	p.M269T/R	RHO GEF	NS
		p.R552/G/S/K	Allosteric site	
		p.E848K	RAS GEF	
Ubiquitin	*CBL*	p.Y371H/C/N	RING	NSLD
	*LZTR1*	p.G248R	Kelch	NS

The table presents information about the most frequent recurring changes that occur in known RASopathy genes and specifies the protein domain where the alteration happens. The selection criteria were based on the clinical outcome (phenotype) of the variants. The acronyms used in the table include CFC, cardiofaciocutaneous syndrome; CS, Costello syndrome; NS, Noonan syndrome; NS-LAH, Noonan syndrome with loose anagen hair; NSLD, Noonan syndrome-like disease; NS-ML, Noonan syndrome with multiple lentigines.

### *PTPN11* (protein-tyrosine phosphatase, nonreceptor-type 11)

*PTPN11* is the most common gene associated with NS and accounts for approximately half of all cases. The *PTPN11* gene (19) has three domains: the more commonly mutated N-amino terminal src-homology 2 (N-SH2) and phospho-tyrosine phosphatase (PTP) domains, and the C-amino terminal src-homology 2 domain (C-SH2) and carboxy-terminal tail (5, 6). *PTPN11* codes for protein SHP-2 which is involved in semilunar valvulogenesis, hemopoietic cell differentiation, and mesodermal patterning (20–23). SHP-2 also regulates the cell proliferation, migration, or differentiation processes during the developmental stage (24) and is widely expressed in several tissues, such as the heart, muscles, and brain. SHP-2 is a pivotal protein in the RAS/MAPK cascade. *PTPN11* mutations were mainly seen in cases with pulmonary valve stenosis in NS1 patients (5, 6).

### SOS1 (Son of seven less homologs 1)

Mutated *SOS1* (OMIM 182530) is considered the second-most-common genetic aberration associated with NS, causing NS in up to 20% of patients with absent *PTPN11* mutation (25). Its locus is on the 2p22-p21 region, consisting of 23 exons (26, 27) and coding for multiple domains containing: regulatory histone-like folds domain (HF), Dbl homology domains (DH), and Pleckstrin homology domains (PH); catalytic RAS exchanging motif (REM) and Cdc25 domains; helical linker (HL) relating PH and REM, and the Polyproline region (25, 28). SOS1 is a guanine exchange factor (GEF) with a significant role in the RAS/MAPK pathway (26, 27) and mainly implicated in NS patients with ectodermal defects (25, 28–30) and pulmonic stenosis more than that of PTPN1.

### *KRAS* (kirsten rat sarcoma viral oncogene homolog)

The *KRAS* (OMIM 190070) gene is mapped to the 12p12 region and consists of 6 exons coding for the P loop and switch I and switch II domains (15). Gain of function mutations in *KRAS* causes approximately 5% of NS cases in the absence of the PTPN11 mutations (16, 31).

### *NRAS* (neuroblastoma RAS viral oncogene homolog)

The *NRAS* (OMIM 164790) gene locus on 1p13.2 comprises six coding exons (32). *NRAS* mutations are involved in less than 1% of NS cases (17).

### *RIT1* (Ric-like protein without CAAX motif 1)

The *RIT1* (OMIM 609591) locus on 1q22, consisting of 6 exons, causes hyperactivation of transcription factor *ELK1* in the RAS/MAPK cascade. It is present in 9% of NS cases (18). Prevalence is seen to mainly coexist with cardiac defects such as CHD (94%), HCM (71%), and pulmonic stenosis (65%). This finding was subsequently confirmed by Bertola et al., who found the exact prevalence (9%), and Gos et al., who found a lower mutation rate (3.8%). Those mutation clusters in the G1, Switch I, and more frequently in Switch II domains, were proven to entail a significant activation of the RAS/MAPK pathway by hyper-activating transcription factor *ELK1* (33, 34).

### *RAF1* (v-RAF-1 murine leukemia viral oncogene homolog 1)

*RAF1* (OMIM 164760) locus on 3p25, consisting of 17 exons, codes for protein serine-threonine kinase (35–37) and has three conserved regions. Mutations causing failure of autoinhibition of this gene lead to activation of the RAS/MAPK cascade, causing NS (3%–17% of cases). An association of 80% is found with HCM.

### *BRAF* (V-Raf murine sarcoma viral oncogene homolog B1)

Mutated *BRAF* (OMIM 164757) locus on 7q34 enhances ERK activation (38, 36), causing NS in 1.7%–1.9% of cases.

### *MAP2K*1 (mitogen-activated protein kinase 1)

*MAP2K1* (OMIM 176872), with a locus on 15q22, comprises 11 exons encoding the MEK protein, which activates ERK-MAP (39). Among NS cases without *PTPN11* and SOS1 mutations, 4.2% are caused by mutated MAP2K1 (40).

### *SOS2* (Son of seven less homolog 2)

Mutation of the homolog of *SOS2* (OMIM 601247) locus on 14q21 causes 4% of Noonan cases, closely associated with ectodermal defects like *SOS1* (41).

### *LZTR1* (leucine-zipper-like transcription regulator 1)

The *LZTR1* gene (OMIM 600574) mapped on 22q11.21, consisting of 21 exons, encodes for a protein of the BTB-ketch superfamily, also implicated in neurofibromatosis (42). However, it is not associated with the RAS/MAPK pathway (41).

### *A2ML1* [a-2-macroglobulin (A2m)-like-1]

Mutation of *A2ML1* (OMIM 610627), mapped on 12p13 with 35 exons, comprises 1% of Noonan patients negative for other significant genes (43). *A2ML1* is a member of the a-macroglobulin superfamily, localized in the 12p13 region with 35 coding exons, and is a protease inhibitor upstream of the MAPK pathway (44). Nevertheless, how its mutation affects the MAPK pathway requires further explication.

## Other genes

Recently implicated rare variants in NS include *RASA2, MAP3K8*, and *SPRY* (45).

### Neurofibromatosis type 1 (NF1) and cardiac manifestation

Neurofibromatosis (OMIM 162200) is an autosomal dominant genetic disorder caused by a heterozygous mutation of the NF1 gene located on chromosome 17q11.2. NF1 is a multisystem disease impacting the growth and function of various cell types and organs. Early-onset cerebrovascular disease, pheochromocytomas, and cardiovascular disease frequently cause premature death in individuals with NF1. Neurofibromas, the characteristic tumors of NF1, impact approximately 1/2000 live births (46) and can develop within the heart, obstructing blood flow in the heart or major vessels by compression or invasion, leading to hemorrhage. Fortunately, these are rare complications. NF1 encodes the neurofibromin protein, which belongs to the family of GTPase activating proteins (GAPs) and which negatively regulates RAS signaling. Neurofibromin also positively regulates cyclic adenosine monophosphate (AMP) levels (47, 48). Increased cyclic AMP levels have been associated with reduced cell growth, likely through interference with multiple mitogenic signaling pathways. The most common cardiovascular manifestations of NF1 include vasculopathy (49), hypertension (50), and other congenital heart defects (51). Sørensen found myocardial infarction and cerebrovascular accidents at a younger than-expected age in NF1 patients (52). NF1 vasculopathy includes segmental hypoplasia of the abdominal aorta and fibro cellular intimal proliferation. Both contribute to the luminal stenosis (53), aneurysms, the rupturing of which has been known to cause catastrophic abdominal and retroperitoneal hemorrhage and arteriovenous malformations, and is the second leading cause of death in neurofibromatosis patients (54–56). Coronary heart disease occurs at a higher-than-expected frequency compared with that in the general population, with pulmonary artery stenosis representing 25% of these malformations. Hypertension is common among female NF1 patients during pregnancy (57), and the prevalence increases with age. However, it has not been investigated whether NF1-hypertension is just a coincidental finding often discovered during medical evaluation for other reasons. Based on the previous literature, 10%–15% of NF1 patients have CHD (51). Approximately 50% of NF1 individuals with CHD have PVS. Aortic stenosis, aortic coarctation, atrial septal defects (ASD), and ventricular septal defects (VSD) are detected less frequently in NF-CHDs (58–60).

### Genetics of neurofibromatosis type 1

*NF1* is a large and complex gene that carries more than 280 kb of genomic DNA, including 57 constitutive exons and other alternatively spliced exons (61). Now, over 2,800 different *NF1* variants have been identified (62). Genetic testing in NF1 is challenging because of the large number of possible mutations in this large gene. Approximately 5% of patients with NF1 have a complete or near-complete deletion of the NF1 gene. These patients display a more severe phenotype, including earlier onset, large load of neurofibromas, greater likelihood of cognitive deficiency, dysmorphic facial features, increased risk of malignancy, and connective tissue involvement, with joint laxity, hyperextensible skin, and mitral valve prolapse. *ADAP2* gene, which has been considered as a modifier of NF1, involved in cardiac development, is a reliable candidate gene for the occurrence of congenital valve defects (63). Additionally, *CENTA2* and *JJAZ1* are two possible candidates for the cardiovascular malformations (64).

### Sex dimorphism in RASopathy-induced cardiomyopathy in NS and NF1

The relationship between RASopathies and sex dimorphism is controversial, complex, and likely influenced by many factors. Studies have suggested that males with NS may be more likely to have more severe cardiac manifestations, including a higher incidence of hypertrophic cardiomyopathy and aortic valve stenosis, compared to females with NS (3, 35, 65). Similarly, few studies indicated that males had a higher incidence of pathogenic variants in the *RAF1* gene, a less common genetic mutation associated with NS (66–69). However, other studies suggested no significant sex differences in the prevalence or severity of cardiac abnormalities in NS patients (16, 70). A retrospective cohort of 412 children with NS by Romano et al. found that female patients had a higher prevalence of pulmonary valve stenosis and a higher incidence of cardiac surgery compared to male patients. These female patients also indicated a higher incidence of composite cardiovascular events compared to male patients (71).

There is limited evidence regarding sex differences in cardiac manifestations of NF1 (72). But a recent study investigated sex differences in cardiac function in NF1 patients with Left Ventricular (LV) dysfunction and found that males had significantly lower Left ventricular ejection fraction (LV EF) and more severe LV dysfunction than females. In addition, males had a higher incidence of LV remodeling and a higher risk of sudden cardiac death than females (73). Similarly, individuals with NF1 found that males were more likely to have cardiac abnormalities than females and that males had a higher incidence of pulmonary stenosis and atrial septal defects (51). On contrary, an older study found that females with NF1 may be more likely to have cardiac abnormalities than males (58).

Current observations indicate that there may be some sex differences in the prevalence or severity of cardiac manifestations in RASopathies. These differences are not always consistent across studies and may be influenced by other factors such as age, genotype, and environmental factors. Additionally, many individuals with RASopathies have a normal cardiac function. However, the mechanisms underlying these sex differences are not well understood. One possible explanation is the differential expression of RAS-MAPK pathway genes in males and females, which could affect the development and progression of cardiomyopathy in RASopathies. Another possible explanation is the influence of sex hormones on cardiac function and remodeling, which could interact with the RAS-MAPK pathway and contribute to sex differences in RASopathy-induced cardiomyopathy. Despite the growing recognition of sex differences in RASopathy-induced cardiomyopathy, there is a lack of sex-specific guidelines for the diagnosis and management of cardiac complications in these disorders. This highlights the need for further research to understand the mechanisms underlying sex differences in RASopathy-induced cardiomyopathy and develop sex-specific management strategies to improve outcomes for both male and female patients.

### Age of onset and clinical penetrance of genetic variants in RASopathies

Cardiomyopathy, a common cardiovascular complication in patients with NS and NF1, is caused by genetic mutations in the RAS-MAPK pathway. The age of onset and clinical penetrance of cardiomyopathy differ between NS and NF1. NS typically presents in childhood or adolescence, while NF1 may not present until adulthood. The penetrance of cardiomyopathy is also higher in NS than in NF1. Colquitt et al. in 2014 demonstrated that in NS patients severe HCM has an early onset with an increased risk of long-term morbidities (74). Later many studies confirmed the early onset of HCM (75, 76) as well as pulmonary valve stenosis and arterial septal defect in NS patients (77). In contrast, the prevalence of HCM in NF1 patients was only 2%, with a mean age of onset of 26 years. Also, mutations in the NF1 gene have been associated with a decreased risk of cardiomyopathy (59).

Several genetic variants have been associated with an increased risk of cardiomyopathy in NS and NF1. In NS, mutations in the *PTPN11* and *RAF1* genes have been associated with an increased risk of HCM. Lin et al. in 2000 found that the prevalence of HCM was higher in NS patients with *PTPN11* mutations than in those with *RAF1* mutations (44% vs. 18%). Overall, 9% of the DCM cohort presenting in childhood or adolescence have *RAF1* mutations (59) *PTPN11* had common echocardiography features characterized by pulmonary valve stenosis, while HCM is characterized by *RAF1*. *RAF1* genotypes were shown as prognostic factors, eliciting multiple interventions that may be required for NS patients with severe pulmonary stenosis or myectomy for HCM (77). But a recent study indicated that the proportion of *RIT1* mutation-positive patients who underwent intervention due to cardiovascular disease was significantly higher than that of patients with *PTPN11* mutations (78). A multi-center cohort study to compare the incidence of sudden cardiac death (SCD) and implantable cardioverter-defibrillator (ICD) use between RAS-HCM (*n* = 188) and P-HCM (*n* = 567) patients showed a lower median age for RAS-HCM. Nonarrhythmic deaths occurred primarily in infancy, and SCD primarily in adolescence (79). Another study suggested the possibilities of prenatal RASopathy testing by comparing the genotypic variations from 352 chromosomal microarray negative cases for prenatal RASopathy testing with post-natal cohorts (25 patients with available prenatal information and 108 institutional database genotypes). The study supported the view that a subset of RASopathy genes and variants that are more frequently associated with complex prenatal features such as hydrops/effusions or serious cardiopathy should be considered in the prenatal evaluation (80).

Trametinib, cobimetinib, and binimetinib are examples of medications that have been approved for use in certain tumors to suppress the RAS/MAPK signaling pathway. These medications may benefit NS patients with mutations resulting in gain-of-function alterations in the RAS/MAPK pathway. This has been investigated in mouse models with the *RAF1* mutation, which is present in many NS patients. Mek inhibition during postnatal treatment reversed hypertrophy, restored standard cardiomyocyte size, and lowered fractional shortening toward the target range (81). Since then, there have been several case reports highlighting anecdotal successes with MEK inhibition in NS patients. By now, three groups have described the cases of four patients who, after using trametinib, showed improvement from NS and HCM (82–84). Studies have also shown that arrhythmia and lymphatic abnormalities resolve after starting MEK inhibition treatment (83, 85, 86). While there are some promising early reports of this medical therapy for a patient population for which it is typically believed that the only treatment option is cardiac transplantation, more research is still required in this area (87).

**Figure 3:**
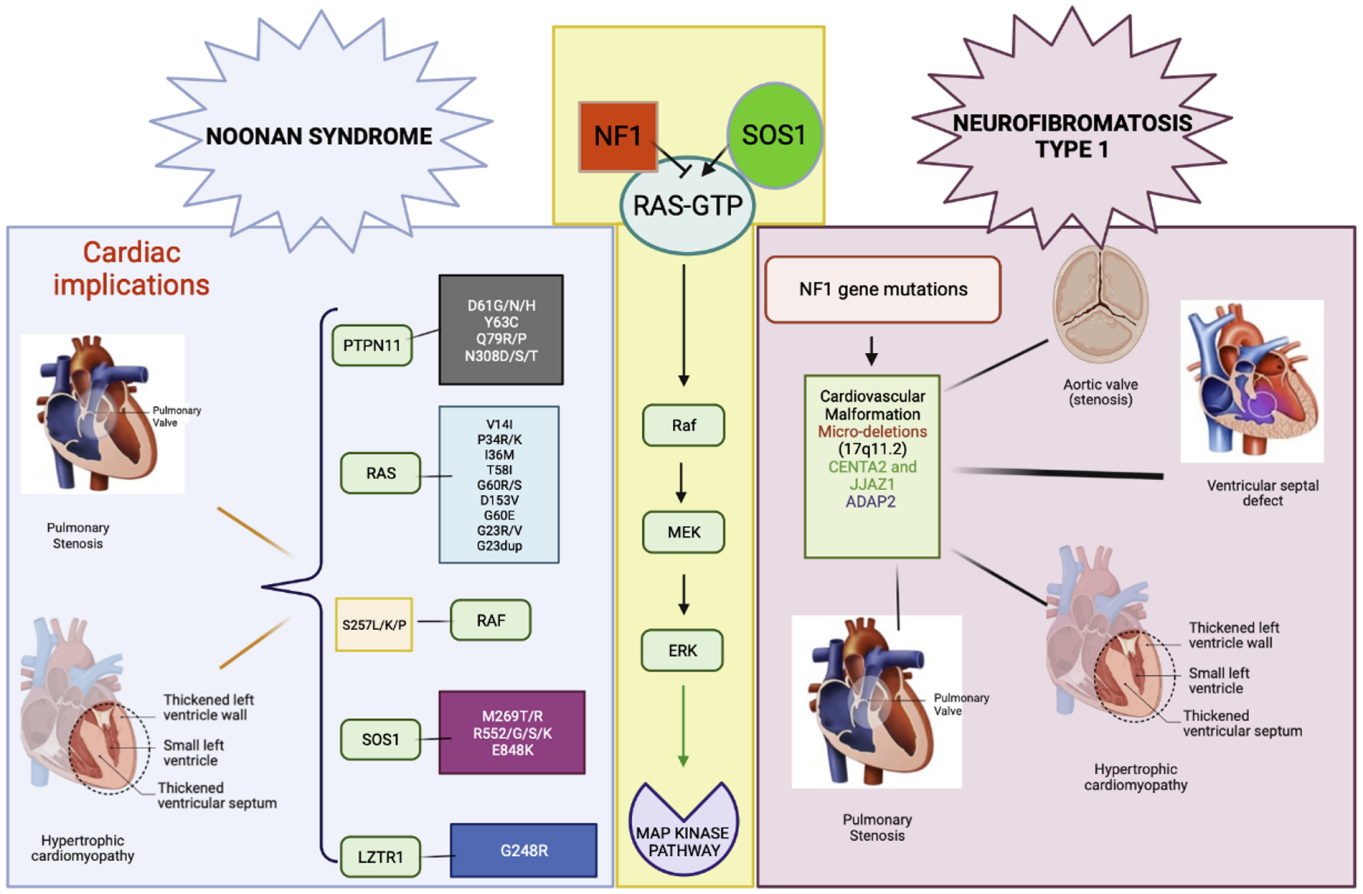
RAS/MAPK pathway gene involvement in NS and NF1. Gene variants and cardiac malformations in NS and NF1.

Our understanding of the molecular basis of RASopathies continues to expand, along with our knowledge of the various clinical manifestations of these disorders, including cardiomyopathy. Figure 3 indicates the involvement of RAS/MAPK pathway genes in NS and NF1. The age of onset and clinical penetrance of cardiomyopathy in NS and NF1 are important factors that can influence the diagnosis and management of these conditions. However, much is still unknown about the mechanisms underlying the development of cardiomyopathy in RASopathies, and further research is needed to identify novel therapeutic targets and improve outcomes for affected individuals. One potential explanation for the variability in age of onset and clinical penetrance of cardiomyopathy in NS and NF1 is the wide range of genetic mutations that can occur within these genes. As we have seen, specific mutations can result in more severe forms of cardiomyopathy, while others may have little to no effect on the heart. Other genetic and environmental factors may also play a role in determining the severity and timing of cardiomyopathy in these individuals. Another possible explanation is that comorbidities, such as hypertension, diabetes, or obesity, can further exacerbate the risk of developing cardiomyopathy in individuals with RASopathies. It is crucial for clinicians to carefully monitor and manage these conditions to reduce the risk of cardiovascular complications in this patient population.

Despite these challenges, genetic testing and imaging technology advances have greatly improved our ability to diagnose and monitor cardiomyopathy in individuals with RASopathies. Identifying specific genetic mutations associated with cardiomyopathy can help guide treatment decisions and improve outcomes for affected individuals.

### De-novo mutations in RAS/MAPK pathway

Since the RAS/MAPK pathway was discovered in humans, the role of these two molecules has been investigated extensively in a wide range of human diseases, including the role of somatic mutations in RAS/MAPK mediated cancer. RAS/MAPK pathway genes are often activated because of germline mutations, referred to as RASopathies, comprising ectodermal and mesodermal development abnormalities and various neoplasia. Interestingly, mutations in RASopathy genes impact different cellular subsets differently, and the phenotype observed in patients varies widely. This phenotype diversity with the same genotype could be due to secondary events modified by epigenetic, environmental, and yet undetermined factors. Recent sequencing technology advances have enabled us to decipher many genotype-phenotype mysteries. A recent study discovered *de novo* variants in PTPN11, RAF1, BARF, SHOC2, RASA1, and HRAS in nine sporadic patients, all of whom had cardiovascular abnormalities along with other Noonan syndromic malformations (88). The above study identified six genes harboring eight *de novo* variants. Two patients with Capillary Malformation-Arteriovenous Malformation (CM-AVM had a novel variant in RASA1. The novel missense variant (NM_002890.2: c.2828T>C, p.Leu943Pro) occurred with an amino acid change from a nonpolar amino acid, leucine (Leu), to another nonpolar amino acid, proline (Pro). This study demonstrates the limitation of phenotype-driven genetics testing and the power of family-based NGS for detecting disorders with a clinically atypical presentation and in severely ill infants with CHDs without known genetic cause. Individuals with RASopathies have been linked to various malformations along with cardiovascular problems. Patients with these illnesses may have improved outcomes when the diagnosis is determined based on phenotype and genotype.

## Conclusion and perspectives

RASopathies are a group of genetic disorders characterized by gene mutations in the RAS/MAPK signaling pathway. These genetic disorders are associated with a broad range of clinical manifestations, including developmental abnormalities, intellectual disabilities, and cardiac defects. Among these disorders' the most common cardiac abnormalities are pulmonary valve stenosis, septal defects, left-sided lesions, and complex forms with multiple anomalies. Early diagnosis and management of these cardiac abnormalities are critical for improving the overall outcome of individuals with RASopathies. With the development of genomic technologies, more details of genetic mutations that result in RASopathies and associated cardiac abnormalities can be identified. The new advancement will provide valuable insights into the pathophysiology of these disorders and may lead to the development of new therapeutics for these debilitating disorders.
